# PIK3CA in Colorectal Cancer

**DOI:** 10.3389/fonc.2014.00035

**Published:** 2014-03-03

**Authors:** Gieri Cathomas

**Affiliations:** ^1^Institut für Pathologie, Kantonsspital Baselland, Liestal, Switzerland

**Keywords:** biomarker, colorectal cancer, PIK3CA, prognosis, prediction, aspirin, adjuvant therapy, EGFR

## Abstract

PIK3CA, the catalytic subunit of PI3K, is mutated in many different tumors, including colorectal cancer (CRC). Mutations of PIK3CA have been reported in 10–20% of CRC, about 80% of mutations found in two hot spots in exon 9 and exon 20. In RAS wild-type CRC, PIK3CA mutations have been associated with a worse clinical outcome and with a negative prediction of a response to targeted therapy by anti-EGFR monoclonal antibodies. However, these findings have not been confirmed in all studies and subsequent more detailed analysis has revealed that these effects may be restricted to mutations in Exon 20. Finally, mutations in PIK3CA may be the long sought biomarker for successful adjuvant therapy with aspirin in patients with CRC. Therefore, PIK3CA mutations appear to be a promising predictive biomarker; however, further data are needed to conclusively define the impact of somatic mutations in the PIK3CA gene for the management of patients with CRC.

## Introduction

Colorectal cancer (CRC) is the third most common malignancy worldwide, affecting more than 1.2 million patients and leads to over 6,000,000 deaths every year (1). Since the seminal paper by Kinzler and Vogelstein in 1996, it is known that the development of CRC is based on an accumulation of hereditary and somatic genetic alterations ultimately leading to the malignant phenotype (2). The development of CRC through adenomatous precursor lesions has further led to secondary prevention strategy of colonoscopy screening. In addition, a large body of work has finally shown that CRC is not a single disease but a heterogeneous group of neoplasms with a different genetic and epigenetic background. A number of molecular classifications have been suggested and the presence of at least three pathways are generally accepted today, including the chromosomal instability pathway (CIN), the microsatellite instability pathway (MSI), and the epigenetic CpG island methylator phenotype (CIMP) (3). Despite this increasing body of knowledge, the therapeutic options in patients with advanced, metastatic disease remains rather restricted, and the prognosis poor. The introduction of new targeted therapeutics, namely the development of antibodies against the epidermal growth factor receptor (EGFR), has raised new hope for successful treating of advanced CRC (4). However, only a subgroup of patients, especially those with a KRAS wild-type tumor, profit from the anti-EGFR therapy (5). Unfortunately, KRAS wild-type is not sufficient to predict clinical response and mutations in other effectors of the KRAS or KRAS related pathway have been anticipated to be predictive for anti-EGFR response. A promising candidate for this prediction is PIK3CA, which may not only be predictive for targeted therapy by anti-EGFR antibodies but also turned out to be probably a positive biomarker for the neoadjuvant use of aspirin.

## Basic Biology of PI3K and PIK3CA

**Figure 1:**
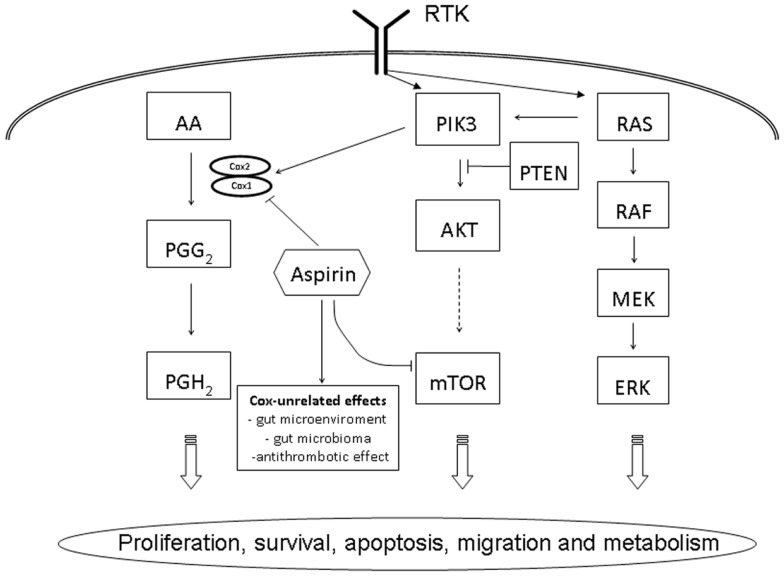
**Schematic overview of PIK3CA related cell signaling pathways in colorectal cancer**. AA, arachidonic acid; AKT, protein kinase B; ERK, extracellular signal-regulated kinase, MEK, MAP kinase kinase; mTOR, mammalian target of rapamycin; PTEN, phosphatase and tensin homolog deleted on chromosome 10; PGG_2_, prostaglandin G2; PGH_2_, prostaglandin H2; PI3K, phosphatidylinositol-3-kinase; RAF, rapidly accelerated fibrosarcoma; RAS, rat sarcoma; RTK, receptor tyrosine kinase.

The phosphatidylinositol-3-kinase (PI3K) belongs to a family of heterodimeric lipid kinases consisting of a regulatory and a catalytic subunit, phosphorylating phosphatidylinositol, an important cell membrane element and second messenger involved in cell signaling (6). Activated by various receptor tyrosine kinases, EGFR, human EGFR 2 (HER2), insulin growth factor (IGF-1R), and platelet derived growth factor (PDGFR), the PI3K promotes and regulates various cellular processes, including proliferation, survival, apoptosis, migration, and metabolism (Figure 1). PIK3CA (phosphatidylinositol-4,5-bisphosphate 3-kinase, catalytic subunit alpha), the catalytic p110-alpha subunit of PI3K, has been described to be commonly mutated in various cancer, including glioblastoma, gastric, breast, ovary, lung, and CRC (7, 8). More than 80% of mutations detected in PIK3CA were reported in two hotspots, the helicase domain of exon 9 (codon 542 and 545) and the kinase domain in exon 20 (codon 1047) (8). Downstream effectors of the PI3K pathway include AKT (protein kinase B), a serine–threonine kinase, directly activated by PI3K and the mTOR (mammalian target of rapamycin), another serine–threonine kinase leading to an increased translation of various mRNAs encoding cell cycle regulators, including MYC and cyclin D1 and a potential target of therapeutic inhibition (9). In the PI3K/AKT/mTOR pathway, the tumor suppressor gene PTEN (phosphatase and tensin homolog deleted on chromosome 10) is a direct antagonist and mutation or loss of PTEN expression has shown to be correlated with a poor outcome in CRC (10, 11).

## Frequency and Prognostic Impact of PIK3CA Mutation in Colorectal Cancer

Roughly, PIK3CA mutation has been reported to be present in 10–20% of CRC. Variation in the frequency has been observed in large population based studies compared to clinical studies (12). In addition, as expected, the technique used to evaluate the PIK3CA mutation has a direct impact on the frequency of mutation observed and using pyrosequencing, generally considered a more sensitive assay, shows higher incidence of PIK3CA mutations (15–18%) (13–15) compared to Sanger sequencing (11–12%) (16–18). Mutations are more commonly found in exon 9 compared to exon 20, usually in a ratio of 3:1 to 5:1 and few tumors (<5%) harbor both mutations (19–21). Interestingly, detailed analysis have shown a gradual decrease of PIK3CA mutation from the proximal (cecum/colon ascendens) to the distal (sigma/rectum) site of colon from 21–25% down to 8–9%, respectively (19, 20). Furthermore, PIK3CA mutated CRC has been associated with a mucinous histological phenotype (15, 19, 20). In contrast to other molecular markers, namely BRAF and KRAS, which are mutually exclusive, PIK3CA mutations have shown in the majority of studies to be significantly associated with KRAS mutation and the loss of MGMT (O6-methylguanine-DNA methyltransferase) expression (15, 19, 20). Less consistent or no correlations where reported for CIMP-H, MSI-H, and BRAF mutations (19, 20, 22). In an early study by Ogino and co-workers analyzing patients with curatively resected CRC stage I–III, mutations in PIK3CA were detected in 18% and reported to be associated with a significant worse outcome. This effect, however, was restricted to tumors with wild-type KRAS (14). Similar results were reported in other patient collectives naïve to anti-EGFR therapy with a frequency of PI3KCA mutation ranging from 12 to 13%; but again, this correlation with poor outcome was restricted to KRAS wild-type tumors (16, 23). Further studies, taking in consideration a broader range of molecular markers, did not confirm these previous data for PIK3CA, being an independent prognostic marker for CRC. Mouradov and co-worker analyzed the disease free survival (DFS) in 822 patients with CRC stage II/III and correlated this data with MSI, CIN, and a number of molecular biomarkers, including PIK3CA (18). In this study, only CIN and MSI were associated with DFS but with none of the molecular biomarkers, including PIK3CA (18). Similarly, in a Scandinavian evaluation, analyzing two study cohorts of 611 patients, PIK3CA did not show any prognostic impact (24). In this study, however, the molecular analysis of PIK3CA was restricted to exon 20, leading to the expected low frequency of 2.2% PIK3CA mutated tumors. The rationale behind this approach is that some studies have suggested that the poor prognosis of PIK3CA is restricted to exon 20 mutations and that the analysis of exon 9 may produce false positive results due to presence of pseudogenes (25, 26). Indeed, in breast cancer, the prognostic impact of PIK3CA has been similarly reported to be restricted to exon 20 (27, 28). This concept is supported by *in vitro* studies showing that mutations in the helical (exon 9) and kinase (exon 20) domain use different and independent mechanisms for cell transformation (29). In addition, the effect of PIK3CA mutation is RAS dependent in the helical but not the kinase domain, which may explain the stronger association of KRAS mutation with exon 9 mutations of PIK3C (19, 22). Taken together, mutation of PIK3CA in CRC may have a slight prognostic impact in anti-EGFR naïve patients; the extent, if present, of this impact, however, especially in respect to different mutations, remains to be clarified.

## PIK3CA as Predictive Marker in Anti-EGFR Therapy

Despite the fact that CRC can curably be treated at early stages, advanced tumors, namely metastatic cancer are associated with a high mortality rate and a 5-year survival of below 10% (30). The introduction of a targeted therapy using monoclonal anti-EGFR antibody, namely panitumumab and cetuximab, in combination chemotherapy or as a single agent, has added a further promising treatment option (4, 31). However, only a subgroup of patients, usually <10% in unselected patients, profit of anti-EGFR antibody treatment (5, 32). Several clinical trials have shown that RAS mutations are the most important negative predictive factor in CRC, primarily mutations in exon 1 and 2 of RAS, but, as recently been shown, also of exon 3 and 4 of KRAS and NRAS, respectively (32, 33). However, even in wild-type RAS tumors, 50–60% of patients do not profit from an anti-EGFR therapy. Based on the well-established pathway of the EGFR receptor, other downstream elements of the direct or associated signaling pathway, including BRAF/MEK/ERK and PIK3/PTEN/AKT/mTOR have been analyzed as potential biomarker (Figure 1). In a first study, analyzing 110 patients with CRC, Sartore-Bianchi and co-workers reported a significant resistance to EGFR-targeted therapy in the 13.6% of PIK3CA mutated cancers (34). The predictive value of PIK3CA mutation in RAS wild-type CRC was supported subsequent by additional studies (35, 36). Interestingly, however, in a study by Prenen and co-workers analyzing 200 chemorefractory patients treated with cetuximab, PIK3CA mutation, detected in 11.5% of tumors, was no predictor of anti-EGFR response (37). Further detailed studies, analyzing PIK3CA mutation of exon 9 and exon 20 separately, may possibly give the explanation for the discrepancy of the predictive value of PIK3CA as a biomarker for anti-EGFR response. In a carefully performed, retrospective study including 743 CRC, de Roock and co-workers describe in KRAS wild-type tumors a significant association of objective response, overall survival, and progression free survival in exon 20 but not in exon 9 mutated tumors (22). As expected, the incidence of exon 20 mutation in PIK3CA was low, i.e., 3.0%, however, the mutation analysis added another 1.3% improvement of anti-EGFR response prediction, similar to the improvement of prediction by testing NRAS (i.e., 1.5%) (22).

## PIK3CA as Biomarker for Adjuvant Aspirin Therapy

Based on several observational studies as well as randomized trials, it has been long considered that aspirin is efficient in preventing colorectal adenomas and cancers (38, 39). This anti-tumor effect is thought to be driven by the inhibition of cyclooxygenases [COX-2, officially called HGNC:9605 or PTGS2 (prostaglandin-endoperoxide synthase 2)], interacting with the arachidonic acid metabolite pathway, however, the detailed mechanism of action is not completely understood [reviewed in Ref. (40)]. This anti-tumor effect has reported to be restricted to patients with cancers showing an over expression of COX-2 demonstrated by immunohistochemistry (41). However, as 60–85% of CRCs has been reported to over express COX-2 (42), immunohistochemistry is considered a less reliable predictive marker for adjuvant aspirin therapy. Due to its side effects, namely gastrointestinal irritation and bleeding, wide spread and unselected chemoprevention by aspirin is not recommended. In addition, more specific COX-2 inhibitors, such as rofecoxib or celecoxib, had to be withdrawn from the market due to their cardiovascular side effects. Therefore, the recent study by Liau and co-workers, reporting an improved survival of CRC patients using regular aspirin in tumors harboring a PIK3CA mutation, has created a lot of interest (13). Using data of two large prospective studies, the Nurses’ Health study and the Health Professionals Follow-up Study, the authors were able to follow 964 patients for a median follow-up time of 153 months. PIK3CA mutations were detected in 16.7% of tumors, and in the patients with mutated cancer, the regular use of aspirin was associated with a reduction of tumor specific and over all mortality of 82 and 46%, respectively. The precise molecular and biological mechanisms of aspirin to the PIK3/AKT/mTOR pathway have to be clarified in detail (Figure 1). *In vitro* studies indicate that the PIK3CA induces the expression of COX-2 (43). In addition, COX unrelated effects, namely the change of the microenvironment and subsequently the intestinal microbioma in the gut as well as the anti-thrombotic effect of aspirin, which may be relevant in the development of metastasis, have to be considered (44, 45). An additional clinical study by Domingo and co-workers, again retrospectively analyzing a large prospective study cohort of 896 patients with CRC, the patients of the VICTOR trial, has confirmed the predictive value of PIK3CA mutation for taking aspirin in an adjuvant setting (46). Interestingly, however, this effect could not be observed in patients taken the specific COX-2 inhibitor rofecoxib. These data indicate that the predictive value of PIK3CA mutation goes beyond COX-2 inhibition and underlies the importance of a COX-independent effect of aspirin in the prevention of cancer development and spread. Despite the promising results reported by these two studies, there is some caveat to express: based on the stratification necessary, i.e., patients with PIK3CA mutated tumors and taking regularly aspirin, the numbers of patients is relatively small in both studies (60 and 45 patients, respectively) and prospective, large scale clinical trials are needed to confirm this data, highly relevant for the future management of patients with CRC.

In conclusion, somatic mutations of PIK3CA are present in 10–20% of CRC, basically confined to exon 9 and exon 20 (or more precisely, these are the exons usually analyzed). The majority of studies are based on pooled data in respect to the exon mutated, but experimental as well as epidemiological evidence point in the direction that mutation in exon 20 but not in exon 9 may be biologically relevant. So far, mutation of PIK3CA as a single prognostic marker seems to have, if some, a minor effect of the overall prognosis of CRC but a small and distinct predictive impact for anti-EGFR therapy in RAS wild-type tumors. In addition, there is strong evidence of the predictive value of PIK3CA mutations for adjuvant therapy using aspirin, however, further data are needed to definitively define the impact of, especially exon specific, PIK3CA mutation in the management of patients with CRC.

## Conflict of Interest Statement

The author declares that the research was conducted in the absence of any commercial or financial relationships that could be construed as a potential conflict of interest.
